# 1,25(OH)_2_VitD3 supplementation enhances suppression of grass pollen-induced allergic asthma by subcutaneous and sublingual immunotherapy in a mouse model

**DOI:** 10.1038/s41598-020-65946-6

**Published:** 2020-06-02

**Authors:** Laura Hesse, Arjen H. Petersen, Joanne N. G. Oude Elberink, Antoon J. M. van Oosterhout, Martijn C. Nawijn

**Affiliations:** 1 University of Groningen, University Medical Center Groningen, Department of Pathology & Medical Biology, Experimental Pulmonary and Inflammatory Research (EXPIRE), Groningen, The Netherlands; 2 Groningen Research Institute of Asthma and COPD (GRIAC), University of Groningen, University Medical Center Groningen, Groningen, The Netherlands; 3 University of Groningen, University Medical Center Groningen, Department of Pathology & Medical Biology, Medical Biology section, Groningen, The Netherlands; 4 University Medical Centre Groningen, Department of internal medicine, Division of Allergy, Groningen, The Netherlands

**Keywords:** Immunotherapy, Immunosuppression, Asthma

## Abstract

Allergen specific immunotherapy (AIT) can provide long-term alleviation of symptoms for allergic disease but is hampered by suboptimal efficiency. We and others have previously shown that 1,25(OH)2-VitaminD3 (VitD3) can improve therapeutic efficacy of AIT. However, it is unknown whether VitD3 supplementation has similar effects in sublingual and subcutaneous immunotherapy. Therefore, we aimed to test VitD3 supplementation in both grass pollen (GP) subcutaneous-IT (SCIT) and sublingual-IT (SLIT) in a mouse model for allergic airway inflammation. To this end, GP-sensitized BALB/c mice received GP-SCIT or GP-SLIT with or without 10 ng VitD3, followed by intranasal GP challenges and measurement of airway hyperresponsiveness (AHR) and inflammation. VitD3 supplementation of GP-SCIT resulted in enhanced induction of GP-specific (sp)-IgG2a and suppression of spIgE after challenge. In addition, eosinophil numbers were reduced and levels of IL10 and Amphiregulin were increased in lung tissue. In GP-SLIT, VitD3 supplementation resulted in enhanced sp-IgG2a levels in serum, enhanced suppression of eosinophils and increased IL10 levels in lung tissue, as well as suppression of AHR to methacholine. These data show that VitD3 increases efficacy of both SCIT and SLIT, by enhancing induction of blocking antibodies and suppression of airway inflammation, underscoring the relevance of proficient VitD3 levels for successful AIT.

## Introduction

Successful allergen-specific immunotherapy (AIT) induces an immunologic state of tolerance towards allergens and represents a disease-modifying treatment for allergic airway diseases, such as asthma and rhinitis^1,2^. AIT is a unique form of therapy wherein allergens are administered via the subcutaneous (SCIT) or sublingual route (SLIT) to render long term relief of symptoms^3,4^. The immunological mechanisms include early mast cell and basophil desensitization, inhibition of eosinophilic inflammation, induction of blocking antibodies, suppression of numbers and activity of allergen-specific Th2 cells and type 2 innate lymphoid cells (ILC2s), increases in regulatory T cells (Treg) and their associated cytokines, such as IL10 and TGF-β1^3–5^. However, AIT regimes are hampered by low efficacy to suppress some clinical features of allergic airway inflammation, such as bronchial hyperresponsiveness. Moreover, the use of allergen vaccines with IgE-crosslinking capacity has safety concerns^6^. To overcome the urgent need for improved AIT efficacy, ideally at lower allergen doses, several strategies have been explored^7^, including the use of adjuvants.

We have previously shown the successful use of 1,25-dihydroxy-vitamin D3 (VitD3) as an adjuvant for AIT in the classical mouse model of ovalbumin-induced allergic airway inflammation^8^. The physiologically active form of VitD3 binds to the VitD3 receptor (VDR), a nuclear hormone receptor, to exert its immunoregulatory properties through induction of tolerogenic dendritic cells (DCs)^9^. VitD3 has been shown to prevent maturation of DCs leading to down-regulation of costimulatory molecules (CD40, CD80, CD86) and enhanced IL10 production^10^, facilitating the generation of adaptive Treg cells^11^.

Recent clinical studies indicate that VitD3 supplementation had limited positive effects on HDM-SCIT treatment, with asthma symptom score as the only improvement compared to control HDM-SCIT treatment^12^. In contrast, VitD3 supplementation of GP-SLIT was reported to suppress nasal and asthmatic symptoms to the control GP-SLIT treated group^13^. The discrepancy between these studies might be due to differences in allergen used (HDM versus GP), duration of treatment (12 versus 5 months) or the route of application of the allergen vaccine. Therefore, a head-to-head comparison of VitD3 supplementation in SCIT versus SLIT will help to evaluate VitD3 as an adjuvant for AIT delivered through either subcutaneous or sublingual application.

We have previously studied SCIT and SLIT using grass pollen (GP) and directly compared the two treatment regimens^14^. Here, we employed this experimental model of side-by-side subcutaneous and sublingual AIT to directly compare the efficacy of VitD3 as an adjuvant between SCIT and SLIT treatments. Both experimental models in BALB/c mice contain two sensitizing intraperitoneal injections comprised of an alum-absorbed GP extract followed by 3 subcutaneous injections for GP-SCIT or 40 sublingual administrations for GP-SLIT with or without 10 ng VitD3. Subsequently, mice are challenged three times with intranasal GP and thereafter, AHR to methacholine is measured as well as serological responses, ear-swelling responses (ESR), eosinophilic inflammation in broncho-alveolar lavage fluid (BALF) and lung, and cytokines after re-stimulation of lung cells. We show that VitD3 supplementation augments induction of blocking antibody responses and leads to enhanced suppression of eosinophilic inflammation and increased production of IL10 in lung tissue in both SCIT and SLIT treatment, while an effect on AHR was observed in SLIT treatment only. These studies underscore the relevance of proficient VitD3 levels for successful AIT and support the potential use of VitD3 as an adjuvant to improve efficacy of both SCIT and SLIT in clinical practice.

## Materials and Methods

### Animals

BALB/cByJ mice (8-9 weeks-old) were purchased from Charles River (L’Arbresle, France) and bred in individually ventilated cages and fed a hypo-allergen GP-free diet (4 kcal/gr, 25% protein, 11% fat, 47% sugars, 5% fibers; AB Diets, Woerden, The Netherlands), which has a theoretical pre-manufacture level of 2,900 IU/kg Vitamin D3. Due to the high sensitivity of vitamin D3 to light, air, heat and humidity, the actual level of Vitamin D3 might alter during storage and usage. Female 7-9-week-old progeny were used for experiments (N = 8). The Institutional Animal Care and Use Committee (DEC) at the University of Groningen approved experiments under license number DEC6209 and all experiments were performed in accordance with relevant guidelines and regulations.

### Induction of allergic asthma and treatment protocols

**Figure 1:**
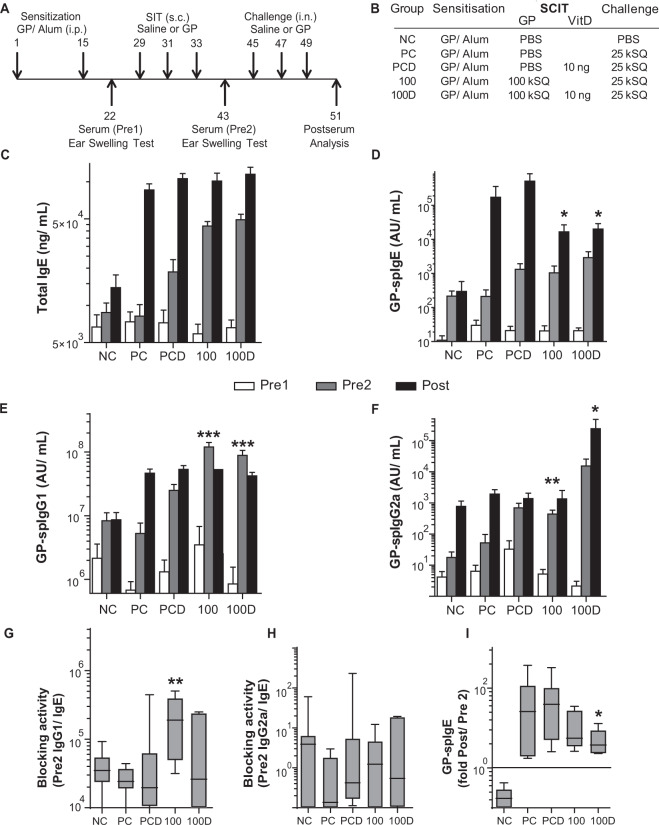
Overview and immunoglobulin response after VitD3-supplemented GP-SCIT treatment. (**A**) Outline of the SCIT protocol. (**B**) Outline of the treatment groups. (**C**) Serum total IgE (ng/mL) taken before SCIT (white bars, Pre1), before challenge (grey bars, Pre2), and after challenge (black bars, Post). (**D**) Serum GP-spIgE (Arbitrary Units (AU)/mL, Pre1, 2, Post). (**E**) Serum GP-spIgG1 (AU/mL, Pre1, 2, Post). (**F**) Serum GP-spIgG2a (AU/mL, Pre1, 2, Post). (**G**) Blocking activity plotted as ratio of GP-spIgG1/GP-spIgE in Pre2 sera. (**H**) Blocking activity plotted as ratio of GP-spIgG2a/GP-spIgE in Pre2 sera. (**I**) Fold induction of GP-spIgE after challenge (Post-sera/Pre2-sera). In Fig. 1C–F, values are expressed as mean ± SEM (n = 8). In Fig. 1G–I, values are expressed in Box-and-whiskers plots (min-max). NC, Negative Control, PBS challenged; PC, Positive Control, GP challenged; PCD, PC with VitD3 in SCIT (10 ng); 100, 100kSQ SCIT; 100D, 100kSQ SCIT with 10 ng VitD3. *P < 0.05, **P < 0.01, ***P < 0.001 compared to PC or PCD respectively (100 vs PC and 100D vs PCD), unless otherwise specified.

All mice received two intraperitoneal injections of 5,000 standardized quality (SQ) units (5kSQ = 8 μg allergen extract of GP (*Phleum pratense*, Phl p; ALK-Abelló, Hørsholm, Denmark) adsorbed to 1.6 mg Alum (Imject Alum, Pierce, USA) in 100 µL Phosphate-buffered Saline (PBS, Figs. 1A,B and 4A,B, S1A,B, and S4A,B). SCIT was performed by three 100 µL injections or SLIT was performed by 40 × 5 µL sublingual administrations, containing either saline or GP with or without 1α,25-dihydroxyvitaminD_3_ (VitD3, Sigma-Aldrich, The Netherlands). Inhalation challenges were administered as droplets of 25kSQ GP in 25 μL PBS after light isoflurane anesthesia. After two days, airway responsiveness was determined, and serum samples, broncho-alveolar lavage fluid (BALF), and lung lobes were stored for further analyses (−80 °C)^7,15^.

**Figure 2:**
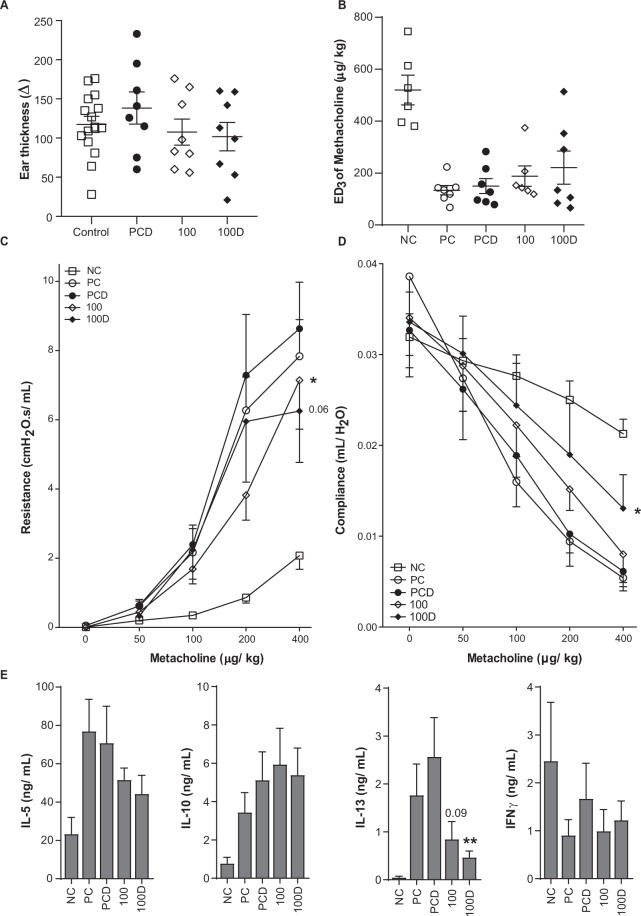
Clinical manifestations after VitD3-supplemented GP-SCIT. (**A**) IgE dependent allergic response plotted as net ear thickness (mm) two hours after GP injection (1kSQ) in the right ear and PBS in the left ear as a control, performed after SCIT. Placebo-SCIT treated mice were plotted together as Controls (NC and PC). (**B**) Effective Dose (ED) of Methacholine, when the airway resistance reaches 3 cmH2O.s/mL. (**C**) Airway hyperactivity (AHR) was measured by FlexiVent and plotted as airway Resistance (R in cmH2O.s/mL) and as (**D**) Airway Compliance (C in mL/cmH2O). (**E**) Net levels of IL5, IL10, IL13, and IFNγ measured in restimulated lung single cell suspensions. Concentrations were calculated as the concentration after restimulation (30ug GP for 5 days) minus unstimulated control (PBS). Absolute values are expressed as mean ± SEM (n = 8). NC: Negative Control, PBS challenged; PC: Positive Control, GP challenged; PCD: PC with VitD3 in SCIT (10 ng), 100: 100kSQ SCIT, 100D: 100kSQ SCIT with 10 ng VitD3. *P < 0.05, **P < 0.01, ***P < 0.001 compared to PC or PCD respectively (100 vs PC and 100D vs PCD), unless otherwise specified.

**Figure 3:**
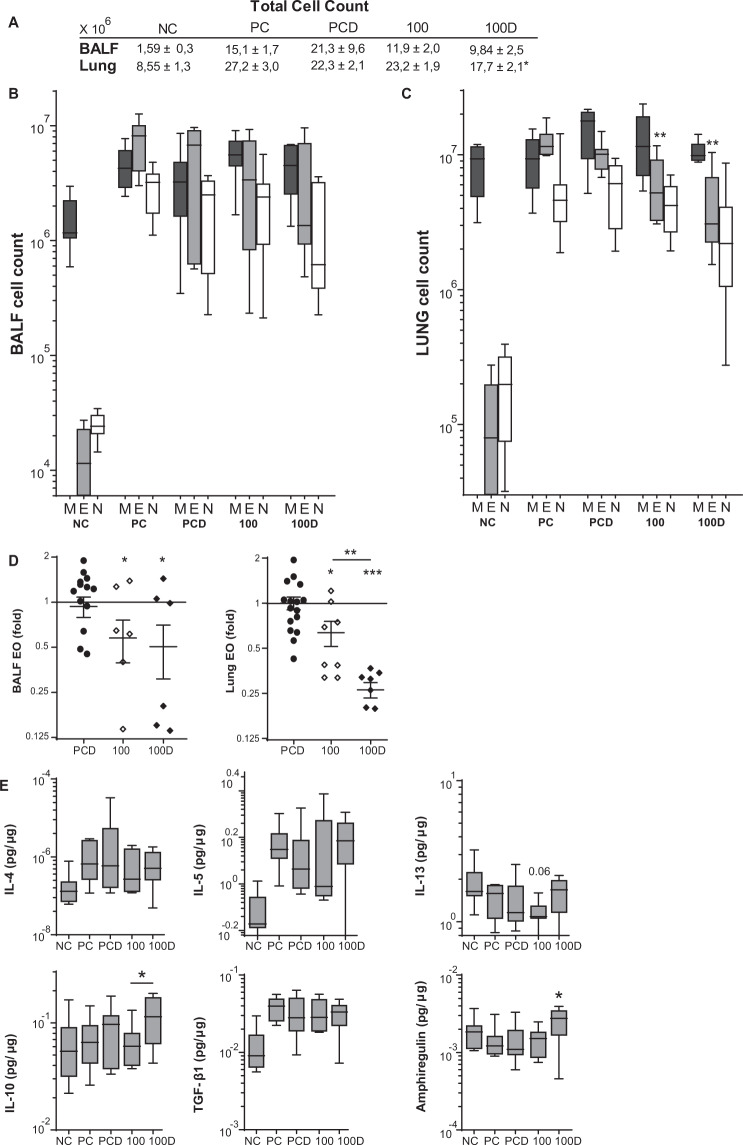
The eosinophilic and cytokine response after VitD3-supplemented GP-SCIT. (**A**) Total cell counts in bronchoalveolar fluid (BALF) and lung single cell suspensions (Lung). (**B**) Differential cytospin cell counts in BALF and in (**C**) Lung. M, mononuclear cells; E, eosinophils; N, neutrophils. Absolute numbers are plotted in Box-and-whiskers plots (min-max). (**D**) BALF and lung eosinophils, both plotted as ratio of suppression (absolute EO/ average PC EO; mean ± SEM). (**E**) Levels of type 2 inflammatory cytokines IL4, IL5, IL13, regulatory cytokines IL10 and TGF-β1, and amphiregulin in pg/µg protein measured in lung tissue. Absolute values are expressed as mean ± SEM (n = 8). NC: Negative Control, PBS challenged; PC: Positive Control, GP challenged; PCD: PC with VitD3 in SCIT (10 ng), 100: 100kSQ SCIT, 100D: 100kSQ SCIT with 10 ng VitD3. *P < 0.05, **P < 0.01, ***P < 0.001 compared to PC or PCD respectively (100 vs PC and 100D vs PCD), unless otherwise specified.

**Figure 4:**
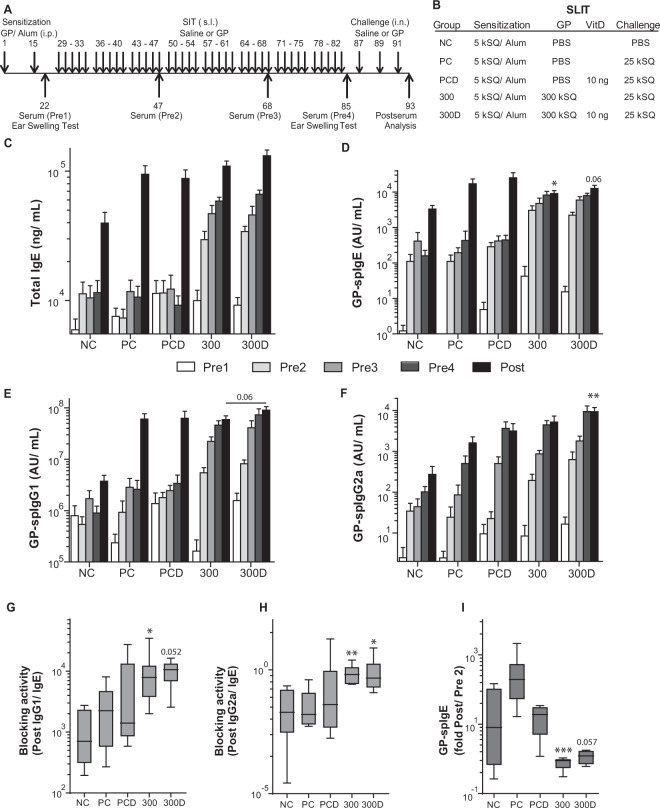
Overview and immunoglobulin response after VitD3-supplemented GP-SLIT. (**A**) Outline of the SLIT protocol. (**B**) Outline of the treatment groups. (**C**) Serum levels of total IgE (ng/mL) taken before SLIT (white bars, Pre1), after 3 weeks of SLIT (light grey bars, Pre2), after 6 weeks of SLIT (middle grey bars, Pre3), before challenge (dark grey bars, Pre4), and after challenges (black bars, Post). (**D**) Serum GP-spIgE (Arbitrary Units (AU)/mL, Pre1-4, Post). (**E**) Serum GP-spIgG1 (AU/mL, Pre1-4, Post). (**F**) Serum GP-spIgG2a (AU/mL, Pre1-4, Post). (**G**) Blocking activity plotted as ratio of GP-spIgG1/GP-spIgE in Post sera. (**H**) Blocking activity plotted as ratio of GP-spIgG2a/GP-spIgE in Post sera. (**I**) Fold induction of GP-spIgE after challenge (Post-sera/Pre2-sera). In Fig. 1C–F, values are expressed as mean ± SEM (n = 8). In Fig. 1G–I, values are expressed in Box-and-whiskers plots (min-max). NC, Negative Control, PBS challenged; PC, Positive Control, GP challenged; PCD, PC with VitD3 in SLIT (10 ng); 300, 300kSQ SLIT; 300D, 300kSQ SLIT with 10 ng VitD3. *P < 0.05, **P < 0.01, ***P < 0.001 compared to PC or PCD respectively (300 vs PC and 300D vs PCD), unless otherwise specified.

### The ear swelling test: early phase hypersensitivity

Before and after SIT treatments, an ear-swelling test (EST) was performed to evaluate the early phase response to GP to test for allergic sensitization. Herein 10 μL of PBS is injected intradermal in the left ear as a control and 1kSQ of GP in 10 μL is injected in the right ear of mice under isoflurane/oxygen anesthesia^14,15^. After 2 h, ear thickness was measured using a digimatic force-micrometer at 0.5 N ( ± 0.15 N, Mitutoyo, Japan) and the net GP-induced swelling (Δ, in µm) was calculated by subtracting the thickness of the left ear from the right ear.

### Airway responsiveness

By measuring airway resistance (R in cmH_2_O.s/mL) in response to intravenous administration of increasing doses of methacholine (Sigma-Aldrich) the airway responsiveness was assessed. Next, lung compliance (C in mL/H_2_O) was examined as a measure of the comparative stiffness of the lung. In short, anesthetized mice were tracheotomized, cannulated through the jugular vein, and attached to a small animal ventilator; the FlexiVent (SCIREQ, Canada), and ventilated (280 breaths/minute) with a tidal volume of 10 mL/kg, pressure limited at 300 mmH_2_O^15,16^. In response to increasing dosages of methacholine, the airway resistance was calculated from the pressure response to a 2-second pseudorandom pressure wave. In analyzing the peak resistance and peak compliance, all values with a coefficient of determination (COD)-value below 0.85 were excluded. Moreover, responsiveness was expressed as the effective dose (ED) of methacholine required to induce a resistance of 3 cmH_2_O.s/mL (ED_3_).

### Evaluating inflammation in BALF

Lungs were lavaged with 1 mL PBS containing 5% Bovine Serum Albumin (BSA, Sigma Aldrich, Zwijndrecht, The Netherlands) and a cocktail of protease inhibitors (Complete mini tablet; Roche, Germany), directly after AHR measurements. Subsequently, four lavages were performed with 1 mL non-supplemented PBS. After centrifugation (500 × g, 4 min), the cell-free supernatant of the first mL was stored as BALF (in *duplo*, −80 °C). The cells from the first mL were added to the cells from the 4 mL PBS lavages and counted using the Z2 coulter particle count and size analyzer (Beckman Coulter, Woerden, The Netherlands).

Cytospin preparations of the BALF and Lung cells were stained with Diff-Quick (Merz&Dade, Dudingen, Switzerland) and 300 cells per cytospin were evaluated and differentiated into mononuclear cells (M), neutrophils (N), and eosinophils (E) by standard morphology.

### Analysis of T cell responses: restimulation of lung single cell suspensions

The left lobes of the lungs were removed, minced and digested for 1.5 h at 37 °C in 2 mL of RPMI1640 (Lonza, Breda, The Netherlands) containing 1% Bovine Serum Albumin (BSA), 4 mg/mL collagenase-A (Roche Diagnostics, Almere, The Netherlands) and 0.1 mg/mL DNAse-I (Roche Diagnostics). Next, lung cells were forced though a 70μm cell strainer (Falcon, Lelystad, The Netherlands), suspended in lysis buffer, washed and suspended again in 10 mL RPMI1640 containing 1% BSA. Total cell counts were established using the Beckman Coulter Counter Z2. Lung cells (5 × 10^5^cells/well) were cultured in RPMI1640 supplemented with 5% Fetal Calf Serum (FCS, Lonza, Breda, The Netherlands), 2 nM Ultra-GlutaMAX (Life Technologies, Bleiswijk, The Netherlands), 100EU penicillin, and 100ug/mL streptomycin in a 96-wells plate (Greiner BioOne, Hannover, Germany) in the presence of 0 μg or 30 μg of GP per well. All samples were stimulated in triplicate for 120 h (CO2 incubator, 37 °C) and the supernatant was collected and stored (−80 °C). ELISA determined concentrations of IL5, IL-10 and IL13, according to the manufacturer’s instructions (BD Pharmingen, San Diego, CA). The lower detection limits of the ELISAs were 32 pg/mL for IL5, 10 mg/mL for IL-10, and 15 pg/mL for IL13.

### Measurement of GP-specific Immunoglobulins in serum

Blood was collected in MiniCollect serum tubes (Greiner Bio-One, Alphen a/d Rijn, The Netherlands) at several time points via orbital puncture (pre-sera) and after the experiment via the vena cava inferior (post-sera, Figs. 1A and 4A)^14^.

Briefly, for GP-spIgE and GP-spIgA, NUNC MaxiSorp flat-bottom 96-well plates (Sigma, MO) were coated with 1 μg/mL anti-mouse IgE or IgA (BD Pharmingen) in PBS (overnight, 4 °C), washed five times (wash buffer; PBS 0.05% Tween-20), blocked using 3% skimmed milk powder (ELK, Campina, Amersfoort, The Netherlands) in ELISA buffer, and sera samples (diluted 1:8 in PBS 1%BSA) were incubated for 2hrs (room temperature). Then, the plates were incubated with 100 μL 1:100 biotin labeled GP in PBS 1%BSA (homemade, see below) for 1.5hrs, washed five times and incubated for 1 h with Streptavidin-Horseradish Peroxidase (1:200, R&D Systems). Again plates were washed, following which SigmaFast-OPD substrate (Sigma-Aldrich) was added and incubated for 8 min. The reaction was stopped by adding 75 μL of 1.8 M H_2_SO_4_. Optical density (OD) values were measured at 490 nm and analyzed using a classic logit-log transformation model.

For GP-spIgG1 and GP-spIgG2a, plates were coated using 10 μg/mL rough extract GP, blocked using 3%BSA in ELISA buffer, incubated with sera samples (1:300,000 for GP-spIgG1 and 1:100 for GP-spIgG2a), and labeled using biotinylated anti-mouse IgG1 or –IgG2a (1 μg/mL, BD Pharmingen). Concentrations were calculated according to the standard curve (using reference serum) and the results are expressed as arbitrary unit (AU)/mL.

Biotinylation of GP extract was performed using EZ-link Sulfo-NHS-LC-Biotin according to the manufacturers operating instructions (Thermo Scientific) and using a Slide-A-Lyzer cassette (3.5 K MWCO, Thermo scientific) for purification by dialysis overnight.

### Analysis of cytokine levels in lung tissue

The right superior lung lobe was used for measurement of total protein content and a cytokine profile. First, lungs were weighed, homogenized and dissolved in Luminex buffer (weight to volume ratio 1:5) and the total protein content was measured using a BCA protein assay according to manufacturer’s protocol (Thermo Scientific, USA). Concentrations of IL4, IL5, IL-10, IL13, IL-17, IL33, IFNγ, Eotaxin (CCL11), TARC (CCL17), and MIP3α (CCL20) were measured using a multiplex Mouse Cytokine/Chemokine Magnetic Bead Panels (MILLIPLEX Map Kit; Merck Millipore, Germany) according to manufacturer’s protocol. Plates were analyzed using a MAGPX1023 4002 with Luminex xMAP technology.

### Statistical analysis

All data are expressed as mean ± SEM. The Mann-Whitney *U* Test was used to analyze the results, and *p* < 0.05 was considered significant. Within the ELISA data, an AU-value which was more than three times the interquartile (IQ) range higher than the upper Q or more than three times the IQ range lower than the lower Q was considered to be an extreme outlier and was removed for further analysis. Within the AHR measurements, to compare the entire curve between groups, a generalized estimated equation (GEE) analysis was used, using SPSS Statistics 20.0.0.2^17^.

## Results

### VitD3 supplementation enhances specific IgG2a responses induced by GP-SCIT

Previously, we developed an experimental mouse model for GP-SCIT and GP-SLIT using a single allergen extract, effectively allowing side-by-side comparison^14^. Here, we aimed to study the efficacy of 10 ng VitD3 supplementation in GP-SCIT and GP-SLIT for suppression of asthmatic manifestations upon GP challenges (Figs. 1A,B and S1A,B). We first evaluated the effect on VitD3 on GP-SCIT treatment. To assess GP-SCIT induced immunoglobulin responses, we measured total IgE, GP-spIgE, GP-spIgG1, and GP-spIgG2a in sera taken before SCIT (white, Pre1), before allergen challenges (grey, Pre2), and after challenges (black, Post, Figs. 1C–F and S1C–F). As previously observed, GP-SCIT injections induced increases in total and GP-specific IgE, as well as increased levels of sp-IgG1 and sp-IgG2a. Upon subsequent GP challenges, GP-SCIT groups displayed a blunted IgE response compared to untreated controls (Figs. 1C,D and S1C,D). Supplementation of GP-SCIT with VitD3 did not alter IgE or IgG1 responses, but induced a strongly increased sp-IgG2a response (Figs. 1C–F and S1C–F).

Clinical efficacy of SIT is associated with the blocking capacity of the spIgGs, while symptom score in allergic asthma is inversely correlated to the ratio of spIgG over spIgE, indicating the relevance of IgG response during SIT^16^. We used the ratios of GP-spIgG1/GP-spIgE and GP-spIgG2a/GP-spIgE as a measure of blocking capacity after GP-SCIT. These ratios were unaffected by VitD3 supplementation (Figs. 1G,H and S1G,H). In addition, we were unable to detect a significant reduction in the fold increase of GP-spIgE levels induced by challenges (GP-spIgE Post/ Pre2) in the SCIT groups, whereas in the VitD3 supplemented GP-SCIT group (100D), we did observe a significant reduction in GP-spIgE as compared to the VitD3 supplemented positive controls (Fig. 1I). Finally, we measured GP-spIgA in BALF and sera taken after challenges (Post) and found that GP-SCIT injections induced increases of GP-spIgA levels in both sera and bronchoalveolar lavages, although VitD was unable to alter those responses (Fig. S1J,K).

### VitD3 supplementation does not enhance suppression of ear swelling or AHR to methacholine

To evaluate the effects of VitD3 supplementation of GP-SCIT on clinically relevant parameters of our experimental model of airway inflammation, we performed an ear swelling test (EST) by intradermal GP injection before and after SCIT treatment in GP-sensitized mice. GP-SCIT did not induce significant suppression of ear swelling after GP challenge, irrespective of VitD3 supplementation (Figs. 2A and S2).

Next, we measured airway hyperresponsiveness (AHR) in response to increasing dosages of methacholine and calculated the dose of methacholine required to induce a resistance of 3 cmH2O.s/mL (ED3; Figs. 2B and S2B). We did not observe a significant increase of the ED3 after GP-SCIT treatment with or without VitD3 supplementation. Next, we compared airway resistance across the entire methacholine dose-response curve and found that GP-SCIT treatment significantly reduced airway resistance compared to the Sham-treated control groups (Figs. 2C and S2C). No effect of VitD3 was observed on this suppression of AHR by GP-SCIT. However, the VitD3 supplemented GP-SCIT resulted in increased compliance in these invasive lung function measurements as compared to the VitD3 supplemented Sham-treated control group (PCD, Figs. 2D and S2D).

To appraise effects of AIT on Th2 driven inflammation, we assessed cytokine levels in lung cell suspensions restimulated *ex vivo* with GP extract (Figs. 2E and S2E). Here, we observed that GP-SCIT-treated mice had significantly reduced IL13 production after *ex vivo* GP stimulation of lung cells, which was a trend only in the GP-SCIT treated group, but reached significance in the VitD3 supplemented GP-SCIT group.

### Suppression of eosinophilic responses after VitD3 supplemented GP-SCIT

To assess suppression of airway inflammation by GP-SCIT, we compared eosinophil numbers in BAL and lung, and cytokine levels in lung tissue homogenates (Figs. 3A–E, and S3). We observed a reduction of lung tissue eosinophil numbers after GP-SCIT treatment (Figs. 3A-C and S3A–C), with the lowest numbers in the VitD3 supplemented group. To compare the effect of VitD3 supplementation on GP-SCIT, we calculated fold reduction in eosinophils of GP-SCIT treated groups with and without VitD3 supplementation relative to their respective Sham-treated groups. Here, we observed an enhancement of the suppression in eosinophil numbers in lung tissue after GP-SCIT by VitD3 supplementation (Figs. 3D and S3D).

Next, we analyzed cytokine levels in lung homogenates after challenges and observed that levels of the type-2 cytokines IL4, IL5 and IL13 were not affected by GP-SCIT treatment (Figs. 3E and S3E). Although no induction of IL10 or TGF-β was observed in GP-SCIT groups, VitD3 supplemented GP-SCIT mice displayed a significantly increased level of IL10 compared to the control GP-SCIT group. Furthermore, only the VitD3 supplemented GP-SCIT group displayed increased levels of amphiregulin in lung tissue after GP challenges when compared to the supplemented positive controls (Fig. 3E).

### VitD3 supplementation enhances specific IgG responses induced by GP-SLIT

Next, we analyzed the effect of VitD3 supplementation on GP-SLIT (Figs. 4A–I and S4). To evaluate the GP-specific immunoglobulin responses during the 14-week treatment protocol^14^, serum was collected at five time points (Figs. 4A,B and S4A,B). We observed a marked and progressive increase in total and GP-spIgE as well as in spIgG1 and spIgG2a during the 8 weeks of GP-SLIT treatment (Figs. 4C-F and S4C–F). Upon subsequent allergen challenges, GP-spIgE responses were blunted in the GP-SLIT treated groups compared to Sham-treated controls, leading to lower levels of spIgE after GP challenges in GP-SLIT treated groups (Figs. 4C,D and S4C,D). Supplementation of GP-SLIT with VitD3 induced a trend towards higher spIgG1 and significantly increased levels of spIgG2a compared to GP-SLIT treated mice in the absence of VitD3 (Figs. 4E,F and S4E,F).

VitD3 supplementation had no effect on the ratios of GP-spIgG1/GP-spIgE and GP-spIgG2a/GP-spIgE after GP-SLIT, used as a measure of blocking capacity (Figs. 4G,H and S4G,H). Furthermore, we observed a striking decrease in fold induction of GP-spIgE by allergen challenges, reflecting the blunted IgE response in the SLIT treated groups, but no effect of VitD3 supplementation was observed (Figs. 4I and S4I). Finally, we measured GP-spIgA in BALF and sera taken after challenges (Post) and found that GP-SCIT injections induced increases of GP-spIgA levels in both sera and BALF (S4J,K). Moreover, addition of VitD in GP-SCIT resulted in a significant increase of GP-spIgA levels after challenges when compared to the unsupplemented GP-SCIT group (Fig. S4J,K).

These data indicate that GP-SLIT treatment induced enhanced blocking, GP-specific immunoglobulin responses while providing a significant decrease of GP-spIgE after challenges. Effects of VitD3 supplementation were detected in the levels of GP-spIgG1 only.

### VitD3 supplementation of GP-SLIT reduces ear swelling and airway hyperresponsiveness

**Figure 5:**
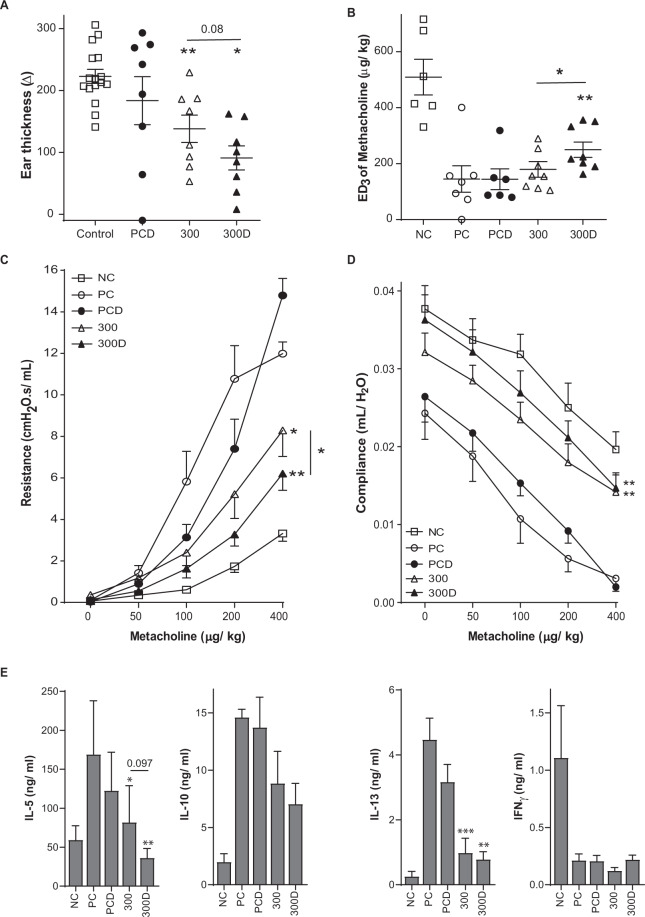
Clinical manifestations after VitD3-supplemented GP-SLIT treatment. (**A**) IgE dependent allergic response plotted as net ear thickness (mm) two hours after GP injection (1kSQ) in the right ear and PBS in the left ear as a control, performed after SLIT. Placebo-SLIT treated mice were plotted together as Controls (NC and PC). (**B**) Effective Dose (ED) of Methacholine, when the airway resistance reaches 3 cmH2O.s/mL. (**C**) Airway hyperactivity (AHR) was measured by FlexiVent and plotted as Resistance (R in cmH_2_O.s/mL) and as (**D**) Compliance (C in mL/cmH_2_O). (**E**) Net levels of IL5, IL10, IL13, and IFNγ measured in restimulated lung cell suspensions. Concentrations were calculated as the concentration after restimulation (30ug GP for 5 days) minus unstimulated control (PBS). Absolute values are expressed as mean ± SEM (n = 8). NC: Negative Control, PBS challenged; PC: Positive Control, GP challenged; PCD: PC with 10 ng VitD3 in SLIT, 300: 300kSQ SLIT, 300D: 300kSQ SLIT with 10 ng VitD3. *P < 0.05, **P < 0.01, ***P < 0.001 compared to PC or PCD respectively (300 vs PC and 300D vs PCD), unless otherwise specified.

Next, we assessed the effect of GP-SLIT on the early-phase response to intradermal GP injections in the ear. Ear swelling was reduced in GP-SLIT treated groups as compared to the sham treated controls. We observed a trend towards increased suppression of ear swelling in the VitD3 supplemented GP-SLIT group as compared to its unsupplemented control (Figs. 5A and S5A).

To measure the effect of GP-SLIT treatment on AHR to methacholine, we measured airway resistance (R) and compliance (C) and calculated the ED_3_ values (R of 3 cmH_2_O.s/mL) in all experimental groups (Figs. 5B–D and S5B–D). The ED3 values were significantly increased only in VitD3 supplemented GP-SCIT treated mice, indicating a reduced sensitivity to methacholine (Figs. 5B and S5B). Indeed, the VitD3 supplemented GP-SLIT group displayed a significantly reduced AHR also when directly compared to GP-SLIT treatment alone (Figs. 5C and S5C). Both VitD3 GP-SLIT as well as GP-SLIT alone showed a significant improvement of lung compliance when compared to their sham-treated controls, however no differences between the groups were detected (Figs. 5D and S5D). Restimulation of lung cell suspensions with allergen extract *ex vivo* to detect allergen-induced cytokine responses revealed marked suppression of allergen-induced IL5 and IL13 production in GP-SLIT mice (Figs. 5E and S5E). VitD3 supplementation of GP-SLIT did not result in augmented suppression of Th2 recall responses *ex vivo*.

### Effects of VitD3 supplemented GP-SLIT on eosinophilic inflammation and cytokine responses

**Figure 6:**
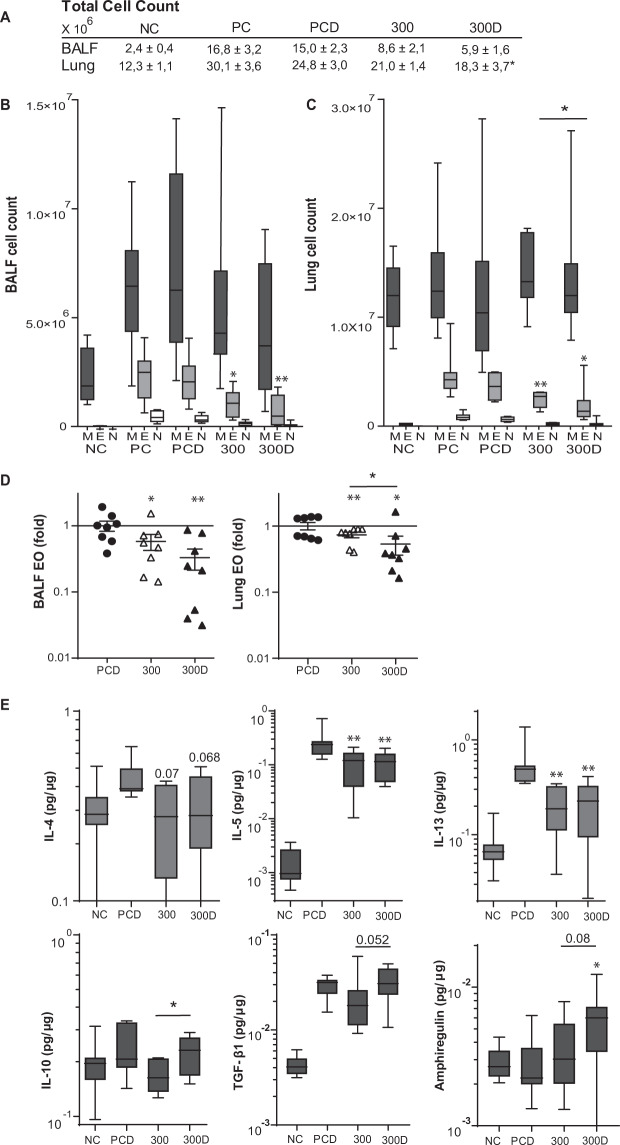
The eosinophilic and cytokine response after VitD3-supplemented GP-SLIT treatment. (**A**) Total cell counts in bronchoalveolar fluid (BALF) and lung single cell suspensions (Lung). (**B**) Differential cytospin cell counts in BALF and in (**C**) Lung. M, mononuclear cells; E, eosinophils; N, neutrophils. Absolute numbers are plotted in Box-and-whiskers plots (min-max). (**D**) BALF and lung eosinophils, both plotted as ratio of suppression (absolute EO/ average PC EO; mean ± SEM). (**E**) Levels of type 2 inflammatory cytokines IL4, IL5, IL13, regulatory cytokines IL10 and TGF-β1, and amphiregulin in pg/µg protein measured in lung tissue of SLIT treated mice. Absolute values are expressed as mean ± SEM (n = 8). NC: Negative Control, PBS challenged; PC: Positive Control, GP challenged; PCD: PC with VitD3 in SLIT (10 ng), 300: 300kSQ SLIT, 300D: 300kSQ SLIT with 10 ng VitD3. *P < 0.05, **P < 0.01, ***P < 0.001 compared to PC or PCD respectively (300 vs PC and 300D vs PCD), unless otherwise specified.

To assess the effect of VitD3 supplementation on airway inflammation, we compared eosinophilic airway inflammation and levels of cytokines in lung tissue after GP-SLIT treatment (Figs. 6A–E and S6A–E). We observed a marked suppression of eosinophil numbers in BAL fluid and lung tissue after GP-SLIT (Figs. 6B,C and S6B,C). Moreover, VitD3 supplementation of GP-SLIT resulted in a significantly reduced number of eosinophils in lung tissue compared to the GP-SLIT group lacking the VitD3 supplementation (Figs. 6B,C and S6B,C). This VitD3 mediated effect on GP-SLIT was also evident when the data were presented as fold reduction in eosinophils of both GP-SLIT treated groups relative to their Sham-treated groups (Figs. 6D and S6D,E).

Finally, we also analyzed cytokine levels in lung homogenates after GP challenges and observed that levels of the type-2 cytokines IL4, IL5 and IL13 were significantly affected by GP-SLIT treatment, but no VitD3 mediated effects were observed (Fig. 6E). Similar findings were observed when other cytokines and chemokines were analyzed after VitD supplemented GP-SLIT (Fig. S6F). However, we were able to show a significant increase of IL10 in the VitD3 supplemented GP-SLIT group compared to the unsupplemented GP-SLIT group. Moreover, levels of TGF-β1 and amphiregulin in lung tissue showed a trend towards an increase in GP-SLIT treatment in the presence of VitD3 (Fig. 6E).

## Discussion

In this study, we investigated whether supplementation of GP-specific immunotherapy with 10 ng VitD3 per administration could enhance the efficacy of both sublingual and subcutaneous administration of the GP allergen extract in suppressing asthmatic manifestations upon GP challenges in an experimental mouse model. We find remarkable similarity in the effects of VitD3 supplementation between GP-SCIT and GP-SLIT treatments: an enhanced GP-specific IgG2a antibody response, suppression of lung tissue eosinophils and increased IL10 levels in lung tissue after GP challenges. In GP-SLIT, we additionally observed an effect of VitD3 supplementation on GP-spIgG1 levels and GP-spIgA levels, as well as on suppression of ear swelling responses and methacholine-induced airway resistance.

Vitamin D insufficiency is widespread, and is thought to contribute to asthma^18^. In some cases, supplementation of VitD3 in clinical studies has resulted in a clear benefit. For instance, VitD3 supplementation during pregnancy reduces the risk of recurrent wheeze and acute respiratory tract infections in early life^18,19^. Moreover, VitD3 supplementation in asthma patients has been shown to reduce the rate of asthma exacerbations requiring treatment with systemic corticosteroids^20^. The mechanism of action is thought to include both steering of the immune system towards a more tolerogenic response, as well as reinforcing the barrier and antiviral properties of the bronchial epithelium^18^. Based on these tolerogenic properties of VitD3, we previously used an experimental SCIT mouse model to show that injection of VitD3 enhanced the therapeutic effects of SCIT in this OVA-driven mouse model for allergic airway inflammation^8^. However, conflicting data have since been obtained in clinical studies using allergen-based SCIT and SLIT treatment protocols^12,13^. These recent studies indicate that VitD3 supplementation had limited positive effects on HDM-SCIT treatment, with asthma symptom score as the only improvement compared to control HDM-SCIT treatment^12^. In contrast, VitD3 supplementation of GP-SLIT was reported to suppress nasal and asthmatic symptoms to the control GP-SLIT treated group^13^. The discrepancy between these studies might be due to differences in allergen used (HDM versus GP), duration of treatment (12 versus 5 months) or the route of application of the allergen vaccine. To resolve whether VitD3 supplementation has the potential to enhance efficacy of both SCIT and SLIT, we here aimed to perform a side-by-side comparison of VitD3 supplementation in SCIT versus SLIT using the same allergen extract in a mouse model of GP-driven allergic airway inflammation.

To our knowledge, this is the first study comparing the adjuvant effects of VitD3 supplementation in GP-SCIT and GP-SLIT treatments in an experimental model for allergic airway disease. Strikingly, and in contrast to previous results using unsupplemented AIT^14^, we here report a prominent Treg cytokine profile in lung tissue after VitD3 supplemented GP-SCIT and GP-SLIT, as demonstrated by the increased levels of IL10 and in SLIT also of TGF-β1. In contrast, clear suppression of Th2 cytokine responses by VitD3 supplementation was not observed. The selective reduction of eosinophils by VitD3 supplementation in GP-SCIT treated mice in absence of a clear suppression of Th2 cell cytokines (Fig. 3D,E), might indicate increased Treg activity, as we have previously shown that Treg depletion prior to allergen challenges mainly affects eosinophilic airway inflammation in SIT-treated mice^21^. However, other sources of IL10 might include regulatory B cells, dendritic cells or innate lymphoid cells^22–25^. Although IL10 and TGF-β1 are not necessarily exclusively produced by regulatory T cells, several studies confirmed the need of IL10 for a successful induction of allergen tolerance^24^. These results are in line with the previously reported biological effects of VitD3 on DCs, which was shown to result in enhanced generation of adaptive Treg cells and IL10 and TGF-β1 production^26,27^. These data support further clinical studies on VitD3 supplementation in allergen-specific immunotherapy treatment.

In literature, supplementation of standard VitD3 levels from 2,000 IU/kg in standard chow to 10,000 IU/kg in supplemented chow or in drinking water resulted in decreased AHR and airway inflammation in mouse model of asthma^28,29^. These studies indicate that systemic levels of VitD do affect airway inflammation and hyperresponsiveness in experimental mouse models. In our study, all mice were fed a standard hypo-allergen diet containing 2,900 IU/kg throughout the experiment, and VitD was applied together with the GP extract, securing high local concentrations at the site of injection, whilst not making a strong contribution to systemic VitD levels (all below 25 ng/mL in serum; data not shown). Therefore, it seems likely that an effect on the phenotype of the local antigen-presenting cell is sufficient to mediate the enhanced effects of VitD3 supplementation on SLIT and SCIT in our experimental mouse models.

In addition, we observe enhanced induction of spIgG1 (SCIT) and spIgG2a (SCIT and SLIT) after VitD3 supplementation. However, we have previously reported that blocking antibodies are not required for suppression of Th2 cell activity and eosinophilic airway inflammation in an experimental OVA-SCIT mouse model^30^. In clinical studies, relevance of IgG4 induction for successful allergen immunotherapy is also under debate^31^. Therefore, further experimental validation would be required to test whether the increased blocking antibody responses associated with VitD3 supplementation contribute to suppression of the allergic response upon allergen re-exposure.

By design, our experimental mouse model mainly captures the allergen desensitization phase of the SIT treatment, while the duration of treatment needed to achieve sustainable suppression of allergic responses seen in patients is not captured in these mouse models^32^. Future improvements on the applicability of the experimental mouse model as a valuable translational research tool could include describing B regulatory cells, (regulatory) innate lymphoid cells and a more detailed overview of cytokine production, like inclusion of IL-35, to deepen our understanding of the mechanisms by which AIT and adjuvants can induce tolerance. In conclusion, we provide evidence that the use of VitD3 supplementation augments induction of blocking antibody responses, and leads to enhanced suppression of eosinophilic inflammation and production of IL10 in lung tissue in both SCIT and SLIT treatments, while in addition an effect on AHR was observed in SLIT treatment.

## Supplementary information

Supplementary Figure S1.

Supplementary Figure S2.

Supplementary Figure S3.

Supplementary Figure S4.

Supplementary Figure S5.

Supplementary Figure S6.

Supplementary Figure legends.
