# Supplementary material for: 1,25(OH)2VitD3 supplementation enhances suppression of grass pollen-induced allergic asthma by subcutaneous and sublingual immunotherapy in a mouse model
Source: Sci Rep. 2020 Jun 2;10:8960. doi: 10.1038/s41598-020-65946-6 (PMC7265339; doi:10.1038/s41598-020-65946-6)
Supplement: Supplementary file 7 — Supplementary Figure legends. [file 41598_2020_65946_MOESM7_ESM.docx]

**1,25(OH)_2_VitD3 supplementation enhances suppression of grass pollen-induced allergic asthma by subcutaneous and sublingual immunotherapy in a mouse model**

Laura Hesse MSc^1,2^, Arjen H. Petersen BSc^3^, Joanne N.G. Oude Elberink^4^, Antoon J.M. van Oosterhout^1^, Martijn C. Nawijn^1,2*^

1: University of Groningen, University Medical Center Groningen, Department of Pathology & Medical Biology, Experimental Pulmonary and Inflammatory Research (EXPIRE), Groningen, The Netherlands.

2: Groningen Research Institute of Asthma and COPD (GRIAC), University of Groningen, University Medical Center Groningen, Groningen, The Netherlands.

3: University of Groningen, University Medical Center Groningen, Department of Pathology & Medical Biology, Medical Biology section, Groningen, The Netherlands.

4: University Medical Centre Groningen, Department of internal medicine, Division of Allergy, Groningen, The Netherlands.

* Corresponding author:

Dr. Ir. M.C. Nawijn, Assistant Professor
Experimental Pulmonary and Inflammatory Research (EXPIRE)
Department of Pathology and Medical Biology
Groningen Research Institute of Asthma and COPD (GRIAC)
University Medical Center Groningen (UMCG)
Hanzeplein 1, internal postcode EA52
9713 GZ Groningen, The Netherlands

Telephone: +31 50 361 0998

FAX number: +31 50 361 9007
E-mail: m.c.nawijn@umcg.nl

**Supplemental Figures**

**Figure S1** Overview, immunoglobulin response and cell counts after GP-SCIT treatment with low dose VitD3. (**A**) Outline of the SCIT protocol in a mouse model of allergic asthma. (**B**) Outline of the treatment groups. GP sensitized mice received either PBS or different doses of GP in SCIT mixed with 10ng VitD3, and were challenged with PBS (Negative Controls) or GP (SCIT treated and Positive Controls). (**C**) Serum levels of total IgE (ng/mL) taken before SCIT (white bars, Pre1), after SCIT (grey bars, Pre2), and after challenges (black bars, Post). (**D**) Serum levels of GP specific IgE (GP-spIgE, Arbitrary Units (AU)/mL). (**E**) Serum levels of GP specific IgG1 (GP-spIgG1, AU/mL). (**F**) Serum levels of GP specific IgG2a (GP-spIgG2a, AU/mL). (**G**) Neutralizing activity plotted as ratio of GP-spIgG1/GP-spIgE in Pre2 sera. (**H**) Neutralizing activity plotted as ratio of GP-spIgG2a/GP-spIgE in Pre2 sera. (**I**) Fold induction of GP-spIgE after challenge (Post-sera/Pre2-sera). In Figure 1C-F, values are expressed as mean ± SEM (n=8). In Figure 1G-I, values are expressed in Box-and-whiskers plots (min-max). NC: Negative Control, PBS challenged; PC: Positive Control, GP challenged; 30, 100, 300: different doses of SCIT treated mice (kSQ), GP challenged. PCD, 30D, 100D: the comparable VitD3 supplemented groups. *P<0.05, **P<0.01, ***P<0.001 compared to PC or PCD respectively (100 vs PC and 100D vs PCD), unless otherwise specified.

**Figure S2** Clinical manifestations after vitamin D-supplemented GP-SCIT treatment. (**A**) IgE dependent allergic response plotted as net ear thickness (mm) two hours after GP injection (1kSQ) in the right ear and PBS in the left ear as a control, performed after SCIT. Placebo-SCIT treated mice were plotted together as Controls (NC and PC). (**B**) Effective Dose (ED) of Methacholine, when the airway resistance reaches 3 cmH2O.s/ mL. (**C**) Airway hyperactivity (AHR) was measured by FlexiVent and plotted as airway Resistance (R in cmH2O.s/mL) and as (**D**) Airway Compliance (C in mL/cmH2O). (**E**) Net levels of IL5, IL10, IL13, and IFNγ measured in restimulated lung single cell suspensions. Concentrations were calculated as the concentration after restimulation (30ug GP for 5 days) minus unstimulated control (PBS). Absolute values are expressed as mean ± SEM (n=8). NC: Negative Control, PBS challenged; PC: Positive Control, GP challenged; 30, 100, 300: different doses of SCIT treated mice (kSQ), GP challenged. PCD, 30D, 100D: the comparable VitD3 supplemented groups. *P<0.05, **P<0.01, ***P<0.001 compared to PC or PCD respectively (100 vs PC and 100D vs PCD), unless otherwise specified.

**Figure S3** The eosinophilic and cytokine response after vitamin D-supplemented GP-SCIT. (**A**) Total cell counts in bronchoalveolar fluid (BALF) and lung single cell suspensions (Lung). (**B**) Differential cytospin cell counts in BALF and in (**C**) Lung. M, mononuclear cells; E, eosinophils; N, neutrophils. Absolute numbers are plotted in Box-and-whiskers plots (min-max). (**D**) Eosinophils in BALF and Lung. (**E**) BALF and lung eosinophils, both plotted as ratio of suppression (absolute EO/ average PC EO; mean ± SEM). (**F**) Levels of type 2 inflammatory cytokines IL4, IL5, IL13, regulatory cytokines IL10 and TGF-β1, and amphiregulin in pg/µg protein measured in lung tissue. Absolute values are expressed as mean ± SEM (n=8). NC: Negative Control, PBS challenged; PC: Positive Control, GP challenged; 30, 100, 300: different doses of SCIT treated mice (kSQ), GP challenged. PCD, 30D, 100D: the comparable VitD3 supplemented groups. *P<0.05, **P<0.01, ***P<0.001 compared to PC or PCD respectively (100 vs PC and 100D vs PCD), unless otherwise specified.

**Figure S4** Overview, immunoglobulin response and cell counts after GP-SLIT treatment with low dose VitD3. (**A**) Outline of the SLIT protocol in a mouse model of allergic asthma. (**B**) Outline of the treatment groups. GP sensitized mice received either PBS or different doses of GP in SLIT mixed with 10ng VitD3, and were challenged with PBS (Negative Controls) or GP (SLIT treated and Positive Controls). (**C**) Serum levels of total IgE (ng/mL) taken before SLIT (white bars, Pre1), after 3 weeks of SLIT (light grey bars, Pre2), after 6 weeks of SLIT (middle grey bars, Pre3), before challenge (dark grey bars, Pre4), and after challenges (black bars, Post). (**D**) Serum levels of GP specific IgE (GP-spIgE, Arbitrary Units (AU)/mL). (**E**) Serum levels of GP specific IgG1 (GP-spIgG1, AU/mL). (**F**) Serum levels of GP specific IgG2a (GP-spIgG2a, AU/mL). (**G**) Neutralizing activity plotted as ratio of GP-spIgG1/GP-spIgE in Post sera. (**H**) Neutralizing activity plotted as ratio of GP-spIgG2a/GP-spIgE in Post sera. (**I**) Fold induction of GP-spIgE after challenge (Post-sera/Pre2-sera). In Figure 1C-F, values are expressed as mean ± SEM (n=8). In Figure 1G-I, values are expressed in Box-and-whiskers plots (min-max). NC: Negative Control, PBS challenged; PC: Positive Control, GP challenged; 300: optimal dose 300kSQ SLIT treated mice (kSQ), GP challenged. PCD, 100D, 300D: the comparable VitD3 supplemented groups. *P<0.05, **P<0.01, ***P<0.001 compared to PC or PCD respectively (300 vs PC and 300D vs PCD), unless otherwise specified.

**Figure S5** Clinical manifestations after vitamin D-supplemented GP-SLIT treatment. (**A**) IgE dependent allergic response plotted as net ear thickness (mm) two hours after GP injection (1kSQ) in the right ear and PBS in the left ear as a control, performed after SLIT. Placebo-SLIT treated mice were plotted together as Controls (NC and PC). (**B**) Effective Dose (ED) of Methacholine, when the airway resistance reaches 3 cmH2O.s/mL. (**C**) Airway hyperactivity (AHR) was measured by FlexiVent and plotted as airway Resistance (R in cmH2O.s/mL) and as (**D**) Airway Compliance (C in mL/cmH2O). (**E**) Net levels of IL5, IL10, IL13, and IFNγ measured in restimulated lung single cell suspensions. Concentrations were calculated as the concentration after restimulation (30ug GP for 5 days) minus unstimulated control (PBS). Absolute values are expressed as mean ± SEM (n=8). *P<0.05, **P<0.01, and ***P<0.005 compared to positive control. NC: Negative Control, PBS challenged; PC: Positive Control, GP challenged; 300: optimal dose 300kSQ SLIT treated mice (kSQ), GP challenged. PCD, 100D, 300D: the comparable VitD3 supplemented groups. *P<0.05, **P<0.01, ***P<0.001 compared to PC or PCD respectively (300 vs PC and 300D vs PCD), unless otherwise specified.

**Figure S6** The eosinophilic and cytokine response after vitamin D-supplemented GP-SLIT. (**A**) Total cell counts in bronchoalveolar fluid (BALF) and lung single cell suspensions (Lung). (**B**) Differential cytospin cell counts in BALF and in (**C**) Lung. M, mononuclear cells; E, eosinophils; N, neutrophils. Absolute numbers are plotted in Box-and-whiskers plots (min-max). (**D**) Eosinophils in BALF and Lung. (**E**) BALF and lung eosinophils, both plotted as ratio of suppression (absolute EO/ average PC EO; mean ± SEM). (**F**) Levels of MIP3α, GM-CSF, IL1α, IL33, KC, IFNγ, IL17, and Eotaxin in pg/µg protein measured in lung tissue. Absolute values are expressed as mean ± SEM (n=8). *P<0.05, **P<0.01, and ***P<0.005 compared to positive control. NC: Negative Control, PBS challenged; PC: Positive Control, GP challenged; 300: optimal dose 300kSQ SLIT treated mice (kSQ), GP challenged. PCD, 100D, 300D: the comparable VitD3 supplemented groups. *P<0.05, **P<0.01, ***P<0.001 compared to PC or PCD respectively (300 vs PC and 300D vs PCD), unless otherwise specified.
